# Coexisting cardiac disease and cervical spinal tuberculosis: Diagnostic challenges and treatment insights

**DOI:** 10.1002/kjm2.12842

**Published:** 2024-05-20

**Authors:** Shu‐Han Hsu, Yoon Bin Chong, Kun‐Bow Tsai, Joon‐Khim Loh

**Affiliations:** ^1^ School of Medicine Kaohsiung Medical University Hospital Kaohsiung Taiwan; ^2^ Division of Neurosurgery, Department of Surgery Kaohsiung Medical University Hospital, Kaohsiung Medical University Kaohsiung Taiwan; ^3^ Department of Pathology Kaohsiung Medical University Hospital, Kaohsiung Medical University Kaohsiung Taiwan

Dear Editor,

Although spinal tuberculosis (TB) cases have been reported for the past several decades, they remain rare in current clinical practice, especially in developed countries.^1^ Cervical involvement is relatively rare.^2^ However, it is even harder to diagnose when its symptoms are atypical and combined heart disease, thus keeping in mind that cervical TB as a differential diagnosis can lead to an early diagnosis. Proper treatment is essential to avoid severe complications, such as deformity, instability, or neurological deficits.^1^ Here, we report an unusual manifestation of cervical spinal tuberculosis masked by coexisting heart disease.

A 49‐year‐old man presented with chest tightness and pain radiating to the neck and interscapular areas for several days. An electrocardiogram and additional laboratory tests indicated the presence of coronary artery stenosis. Subsequently, a percutaneous transluminal angioplasty was successfully performed, during which a bare metal stent was deployed. However, the patient continued to report progressive neck pain post‐procedure despite the normalization of follow‐up cardiac enzyme levels.

Plain cervical spine radiography revealed mild kyphosis and decreased height of intervertebral disc C4‐5 with radiopaque osteophyte formation, which looked more like degeneration related. However, prevertebral soft tissue swelling was also noted. (Figure 1A) Magnetic resonance imaging (MRI) disclosed high signal intensity over C4, C5 vertebral body and C4‐5 disc accompanied with a prevertebral hyperintense lesion extended from level of C1 to C7, which led to a diagnosis of infectious process (Figure 1B,C). Cross‐sectional MR disclosed central extrusion of the C4‐5 disc with compression of the adjacent spinal cord. Due to severe spinal stenosis, surgical management is recommended, and anterior cervical discectomy and fusion were done after draining the pus culture. Trabecular Metal™ Material cage (TM cage, Zimmer Trabecular Metal Technology, Inc., Parsippany, NJ, USA) was used for fusion in C3‐4, C4‐5, and C5‐6.

**FIGURE 1:**
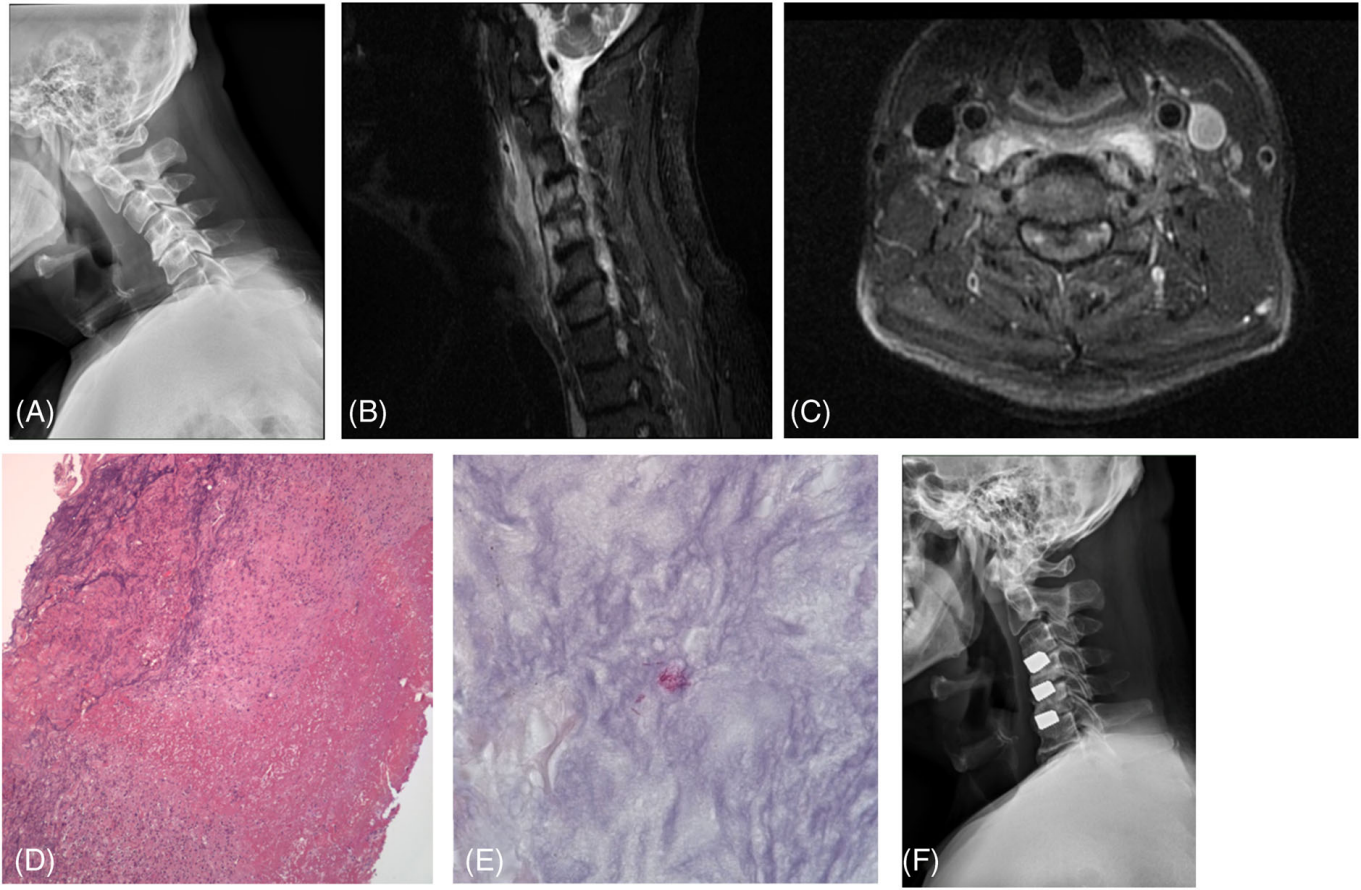
(A) Pre‐operative x‐ray showed degenerated change of cervical spine and swelling prevertebral soft tissue. (B). MRI disclosed edematous change in the cervical 4–5 disc and C4 and C5 vertebral bodies. Prevertebral hyperintense lesions were noted on the T2 weighted image. (C). Cross‐sectional MR image showing central extrusion of the cervical discs with compression of the adjacent spinal cord. (D). Histopathologic examination revealed caseous necrosis with marked inflammatory infiltration (hematoxylin and eosin stain; original magnification, ×10). (E). A few bacilli compatible with mycobacteria were morphologically identified by acid‐fast staining (acid‐fast stain; original magnification, ×100). (F). Follow‐up plain radiograph 8 months after the operation showing the cages instrumentation at cervical 3–4, 4–5, and 5–6 and corrected kyphosis in the lateral view.

Histopathological examination shows necrotizing granulomatous inflammation, composed of central necrotic zone surrounded by epithelioid histiocytes and Langhans type giant cells. Therefore, the diagnosis spinal tuberculosis infection is made (Figure 1D,E). A one‐year, four‐drug anti‐tuberculosis regimen with ethambutol (Epbutol) (400 mg 3 tab every day; Yuseng Chem & Pharm Co. Ltd., Taiwan R.O.C.), pyrazinamide (Pyrazinamide) (500 mg 4 tab every day; PeiLi Pharm Ind. Co. Ltd., Taiwan R.O.C.), and rifampicin plus isoniazid (Rifinah) (300 mg 2 tab every day; Sanofi Co. Ltd.) was initiated. The patient recovered without neurological deficits and no signs of recurrent infection were noted during follow‐up for 1 year.

Given the patient's infectious state, we chose a single‐stage procedure over a two‐stage procedure for several reasons. The initially involved structure was vertebral body rather than disk, which is more common in TB than other pyogenic infections. TB, unlike pyogenic organisms, does not adhere to metals or form biofilms; therefore, the instrumentation is safe even in active disease.^3^ Accordingly, the TM cage was used as the implant material. Several structural characteristics render this material a protective agent against infection. First, polymorphonuclear neutrophil granulocytes and peripheral blood mononuclear cells^4^ are some of the first cells to interact with the implant material and release significantly more cytokines in the presence of TM material. Moreover, the study also concluded that leukocyte activation at the surface of the TM material induces a microenvironment that may enhance local host defense mechanisms, increasing phagocytic, chemotactic, and bactericidal capacity.^4^ Finally, tantalum was believed to have a higher potential to osseointegrate, obliterate the dead space, and prevent organisms from accessing the surface.^5^

In conclusion, the diagnosis of spinal TB is often complicated when a disease with similar symptoms as in this patient is often overlooked; thus, having a differential diagnosis of spinal TB is essential. A one‐stage procedure for spinal TB is a viable treatment, especially using a TM cage due to its property that enhances immune reaction and prevents patients from undergoing a second surgery.

## CONFLICT OF INTEREST STATEMENT

All authors declare no conflict of interest.
